# Impact of communication in healthcare on care’s quality and strategic interventions

**DOI:** 10.1192/j.eurpsy.2026.11718

**Published:** 2026-07-31

**Authors:** A. Sdiri, I. Betbout, M. Harran, H. Zaalouni, M. Ben Mbarek, N. Faouel, A. Ben Haouala, L. Gassab, A. Mhalla

**Affiliations:** 1 Psychiatry Research Laboratory LR05ES10 (Vulnerability To Psychoses), Faculty Of Medicine Of Monastir, University Of Monastir; 2 Psychiatry, Fattouma Bourguiba university hospital, Monastir; 3 UPSAT, Sousse, Tunisia

## Abstract

**Introduction:**

Interprofessional collaboration represents a fundamental component of organizational effectiveness across all sectors. In the context of healthcare, particularly within hospital settings, it is of a greater importance, as the complexity of care delivery requires increasingly sophisticated coordination and communication among diverse healthcare professionals.

**Objectives:**

The aim of this study is to asses the impact of communication among healthcare professionals on care’s quality and to propose strategic interventions.

**Methods:**

This descriptive cross-sectional qualitative study was conducted from January to March 2025 among healthcare professionals working in the emergency and intensive care units of three university hospitals in Tunisia, using a self-administered questionnaire.

**Results:**

The majority of respondents were female (78.4%) with a sex ratio of 0.28, with over half being under the age of 30 (56.8%). Most held the position of nurse (75.2%), among whom 70.2% were senior nurses. Nearly two-thirds had less than five years of professional experience (64.0%) and mainly worked morning shifts (56.8%). A large majority had obtained their degrees after 2006 (86.4%) and worked in intensive care units (57.6%). Most respondents reported working six hours per day (91.2%), and over half were single (55.2%). A majority of respondents (92%) believe that interpersonal communication reduces patient anxiety. Similarly, 80.8% agree that effective communication promotes treatment adherence, while 89.6% think it enhances patient satisfaction. Additionally, 88.8% consider that communication can help reduce conflicts. Over half (59.2%) reported observing improved clinical outcomes as a result of good interpersonal relationships. The most frequently suggested tools to improve communication were interpersonal communication training (59.2%) and fostering a culture of respect and active listening (56.8%). Interestingly, 77.6% of respondents did not believe that regular meetings or debriefings would improve care coordination and continuity. Among those who answered “yes,” the majority (53.6%) felt that holding one meeting per week would be beneficial.

**Conclusions:**

These findings highlight the perceived importance of interpersonal communication in improving various aspects of patient care, including anxiety reduction, treatment adherence, patient satisfaction, and conflict prevention. Strategic efforts should prioritize enhancing interpersonal skills and fostering respectful team dynamics to strengthen communication among healthcare professionals.

**Disclosure of Interest:**

None Declared

